# Investigation of the Prevalence of Chronic Pulmonary Effusion Causes and Response to Treatment (Pleurodesis) in Patients

**DOI:** 10.2174/0118743064336968250113102919

**Published:** 2025-01-16

**Authors:** Alireza Shirzadi, Izadmehr Ahmadinejad, Mojtaba Ahmadinejad, Saeed Hatami, Ali Soltanian, Yasmina Ahmadinejad

**Affiliations:** 1 Department of Surgery, School of Medicine, Alborz University of Medical Sciences, Karaj, Iran; 2 Students' Scientific Research Center, Tehran University of Medical Science, Tehran, Iran; 3 Research Committee, Alborz University of Medical Sciences, Karaj, Iran; 4 Research Committee, Shahid Beheshti University of Medical Sciences, Tehran, Iran

**Keywords:** Pleural effusion, Malignancy, Heart failure, ESR, WBC, HB

## Abstract

**Background:**

Pleural effusion, the pathological accumulation of fluid in the pleural space, is widespread. This study investigates pleural effusion in terms of malignancy among patients referred to tertiary health care centers and evaluates the response rates to different pleurodesis techniques.

**Methods:**

This cross-sectional study enrolled all patients with pleural effusion referred to a tertiary health care center. Laboratory data, including White Blood Cell count and differentiation (WBC), Hemoglobin levels (HB), Erythrocyte Sedimentation Rate (ESR), and biochemical analysis results of the pleural fluid (protein, glucose, and lactate dehydrogenase) were recorded. Data from pleural fluid cytopathological examination, including cell count, cell types, gram staining, and pleural fluid culture, were also documented. Patients undergoing pleurodesis were assessed for response rates, which were categorized as complete, partial, or no response based on clinical and radiological criteria. Collected data were subjected to statistical analysis.

**Results:**

The study investigated 144 patients with chronic pleural effusion, with an average age of 47.59 years. Of these, 97 patients (66%) were male and 47 patients (34%) were female. The most common cause of chronic pleural effusion was malignancy, with a prevalence of 65.9%. Among patients treated with pleurodesis, the overall response rate was 78.4%, with chemical pleurodesis achieving a higher complete response rate (65%) compared to mechanical pleurodesis (55%). Other prevalent causes of chronic pleural effusion, in descending order, included heart failure, liver cirrhosis, chronic kidney failure, and unknown factors.

**Conclusion:**

This study highlights malignancy and chronic heart failure as the predominant etiologies of chronic pleural effusion in a tertiary healthcare setting. Furthermore, it emphasizes the efficacy of pleurodesis techniques, with chemical pleurodesis demonstrating superior outcomes. These findings offer valuable insights into the pathogenesis and management of chronic pleural effusion.

## 1 INTRODUCTION

The pleural space, which develops between the fourth and seventh week of embryonic development [1], plays a crucial role in pleural space homeostasis. The visceral and parietal pleura, originating from the lateral plane mesoderm, are essential components of this space [2]. Derived from embryonic mesoderm, the pleural mesothelium comprises a layer of mesothelial cells that envelop the chest wall and lungs at the parietal and visceral levels. In terms of cytological characteristics, these cells resemble mesothelial cells that cover other body cavities, such as the peritoneum [3,4].

Pleural effusion, arising from altered fluid production, absorption, or a combination of these factors, affects over 1.5 million patients annually in the United States. Key causes include heart failure, pneumonia, and cancer. It becomes clinically evident once fluid exceeds lymphatic absorption capacity [4]. While vital, the pleural space remains an area of ongoing research and debate [5]. Excess fluid accumulation in the pleural space can result from various benign or malignant causes [6]. Specifically, Malignant Pleural Effusion (MPE) denotes effusion marked by the presence of malignant cells. MPE is a common manifestation in patients with metastatic disease, affecting up to 15% of individuals with cancer [7]. It is predominantly associated with lung cancer, followed by breast cancer, lymphoma, gynecological cancers, and malignant mesothelioma. Annually, approximately 150,000 new cases of MPE are diagnosed in the United States, with an additional 100,000 cases in Europe [8]. The overall survival rate for patients with MPE ranges from 3 to 12 months after diagnosis, and common symptoms include shortness of breath, cough, and chest pain [9].

Managing MPE remains challenging due to the paucity of high-quality evidence and heterogeneity in clinical presentations worldwide. Between 30% and 50% of patients with metastatic malignancies show pleural involvement at autopsy, with pleural effusions of varying sizes observed in approximately half of these cases [10]. Despite the global burden of pleural effusion, current knowledge about its etiologies and clinical characteristics is insufficient, particularly in regions with limited research infrastructure [11]. This lack of data limits our ability to develop context-specific diagnostic and therapeutic strategies.

The geographical variation in causes and outcomes of pleural effusion underscores the need for region-specific studies. Understanding the unique epidemiological and clinical patterns in under-researched areas like ours can inform tailored management approaches and enhance global knowledge. Therefore, this study aims to investigate pleural effusion associated with malignancy among patients referred to Shahid Madani Medical Education Center during 2017–2018. By addressing the scarcity of data in this geographical region, we hope to bridge critical knowledge gaps and provide insights that can guide clinical practice and future research.

## 2 MATERIALS AND METHODS

The present study was conducted cross-sectionally. All patients referred with pleural effusion to educational centers from April 2019 to March 2021 were included in the study based on the inclusion and exclusion criteria.

### 2.1 Participant Selection

Patients with pleural effusion referred to educational centers within the specified period were enrolled based on predefined inclusion and exclusion criteria. Diagnosis of pleural effusion had been established in other medical facilities before referral. Patients were referred to the thoracic surgery subspecialist at the study centers for additional measures and treatment. Ethical approval and research council consent were obtained before commencing the study.

### 2.2 Inclusion and Exclusion Criteria

Inclusion criteria included age over 18 years, diagnosis of pleural effusion on imaging, and previous pleurodesis for patients.

Exclusion criteria were established to ensure the study’s focus on patients with chronic pleural effusion suitable for pleurodesis and to avoid confounding variables that could influence outcomes. These criteria included

#### 2.2.1 Unstable Vital Signs at Presentation

Patients in critical condition were excluded as their management priorities differed significantly from those of stable patients with chronic pleural effusion.

#### 2.2.2 Hemothorax

Patients with hemothorax were excluded because the etiology and management of hemothorax are distinct from other types of pleural effusion, often requiring surgical intervention rather than pleurodesis.

#### 2.2.3 Chylothorax (trauma patients)

Patients with chylothorax were excluded due to its specific underlying causes (trauma or lymphatic disruption) and the unique management approaches required, which are beyond the scope of this study.

#### 2.2.4 Loculated Pleural Effusion

Patients with loculated pleural effusion were excluded as loculated effusions often necessitate surgical intervention or fibrinolytic therapy, which differs significantly from the management of free-flowing effusions.

#### 2.2.5 Tuberculosis with Lung Tissue Destruction

Patients with tuberculosis and lung tissue destruction were excluded because tuberculosis-related effusions often require prolonged anti-tuberculous therapy, and their management may extend beyond pleurodesis. Additionally, severe lung destruction may contraindicate pleurodesis.

#### 2.2.6 Insufficient Information in Hospital Records

Patients with incomplete records were excluded to maintain data reliability and integrity.

### 2.7 Data Collection

First, the demographic information of the patients, including age, gender, and presenting symptoms (cough, shortness of breath, *etc*.), was recorded. Radiological information, including type and side of pleural effusion (right/left/both), was recorded. Then, the laboratory information of the patients, including the number of White Blood Cells and their differentiation (WBC), the level of HB and the rate of Erythrocyte Sodium concentration (ESR), and the results of the biochemical analysis of the pleural fluid, including the amount of protein, glucose and lactate dehydrogenase, were recorded. Pleural fluid cytopathological examination results, including the number and type of cells, gram staining, and pleural fluid culture, were recorded.

### 2.8 Data Analysis Method

Sample size estimation was based on similar studies, with consideration for a 95% confidence level, 0.05 error coefficient, and 80% power. The sample size of 150 individuals was calculated using the following formula (43), taking into account the standard variation rate (a/2), standard deviation (σ), and desired error (d).


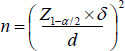


We analyzed the obtained data using SPSS software. SPSS software version 22 was used. Chi-score and one-way ANOVA statistical tests were used and a *p*-value less than 5% was considered significant. The results were presented as a mean and standard interval.

This study was approved by the Research Ethics Board of Alborz University of Medical Sciences (IR.ABZUMS.REC.1399.276).

The methods are stated by STROCSS guidelines. STROCSS (Strengthening the Reporting of Cohort Studies in Surgery) is a set of guidelines specifically developed to enhance the reporting quality and transparency of cohort studies in surgery. These guidelines aim to improve the reproducibility, reliability, and interpretability of surgical research.

## 3 RESULTS

### 3.1 Patient Characteristics

In this study, 144 patients with chronic pleural effusion were investigated. The average age of the patients was 47.59 years, with a standard deviation of 12.13 years, ranging from 18 to 78 years.

### 3.2 Pleural Fluid Culture and Cytology

Pleural fluid culture results were positive in 4 cases (2.8%), while the remaining cases showed negative cultures. Pleural fluid cytology results indicated confirmed malignancy in 56 out of 95 cases (58.9%). The type of pleural effusion was exudative in 99 cases out of 144 patients (68.7%).

### 3.3 Comparative Analyses

The average age and sexual desire of patients with Malignant Pleural Effusion (MPE) were compared to those of other patients using the Independent T-test and Fisher's exact test, respectively. No significant differences were found between the two groups (*p* > 0.05).

### 3.4 Frequency of Pleural Effusion Area and Symptoms

A comparison was made between patients with MPE and other patients using Fisher's exact test to assess the frequency of pleural effusion area and symptoms. The analysis indicated no significant differences between the groups (*p* > 0.05).

### 3.5 Laboratory Findings

Laboratory findings for patients with Malignant Pleural Effusion (MPE) and those without MPE are compared and summarized in Table **1**. Statistical analysis revealed notable differences between the two groups across several laboratory parameters. Patients with MPE exhibited a significantly higher mean white blood cell count (MPE: 11978.4, SD=476.62; non-MPE: 771.39, SD=342.22) (Independent T-test, *p*=0.0001). Hemoglobin levels showed no significant variation between patients with MPE (11.25, SD=3.07) and those without MPE (11.77, SD=3.12) (Mann-Whitney U test, *p*=0.276). Meanwhile, the mean Erythrocyte Sedimentation Rate (ESR) was notably elevated in patients with MPE (38.94, SD=10.89) in comparison to patients without MPE (21.86, SD=9.65) (Independent T-test, *p*=0.0001). Protein levels demonstrated no significant discrepancy between patients with MPE (6.54, SD=0.73) and those without MPE (6.40, SD=0.76) (Independent T-test, *p*=0.276). Patients with MPE exhibited significantly higher Lactate Dehydrogenase (LDH) levels (MPE: 661.59, SD=82.70; non-MPE: 564.25, SD=90.16) (Independent T-test, *p*=0.0001).

Furthermore, laboratory findings pertinent to pleural effusion were analyzed, encompassing protein levels, LDH levels, glucose levels, white blood cell count, and lymphocyte percentage. Mean values and standard deviations were reported for each parameter. Statistical analysis revealed significant differences between patients with MPE and those without MPE across various laboratory findings associated with pleural effusion. Patients with MPE showed markedly higher protein levels in pleural effusion (MPE: 4.21, SD=0.92; non-MPE: 2.52, SD=0.75) (Independent T-test, *p*=0.0001). LDH levels in pleural effusion were substantially elevated in patients with MPE (MPE: 542.76, SD=88.13; non-MPE: 322.68, SD=95.18) (Independent T-test, *p*=0.0001). Glucose levels displayed no significant difference between patients with MPE (112.66, SD=16.70) and those without MPE (114.16, SD=17.57) (Independent T-test, *p*=0.599). Patients with MPE demonstrated notably higher white blood cell counts in pleural effusion (MPE: 5224.03, SD=1207.23; non-MPE: 492.89, SD=91.83) (Independent T-test, *p*=0.0001). The percentage of lymphocytes in pleural effusion was significantly higher in patients with MPE (78.12, SD=11.07) compared to those without MPE (30.22, SD=15.01) (Independent T-test, *p*=0.0001).

### 3.6 Type of Pleural Effusion Prevalence

The prevalence of the type of pleural effusion of patients with MPE (malignant pleural effusion) and other patients are compared in Table **2**, which had significant differences with each other in Fisher's exact test (*p*<0.05).

The table presents the distribution of pleural effusion types among the two groups. In patients with MPE, a vast majority (90.9%) had an exudative pleural effusion, while a minority (11.1%) had a transudative pleural effusion. In contrast, among the other patients, only a small proportion (9%) had an exudative pleural effusion, while the majority (88.8%) had a transudative pleural effusion. The statistical analysis using Fisher's exact test demonstrates a highly significant association between the type of pleural effusion and the presence of malignant pleural effusion (*p* < 0.05). This suggests that the type of pleural effusion can serve as an important indicator in distinguishing patients with MPE from other patients.

### 3.7 Pleurodesis and Treatment Response

Of 95 patients with malignant pleural effusion, 95 cases (100%) underwent pleurodesis, of which 73 (76.8%) patients underwent chemical pleurodesis, and 22 (23.1%) underwent mechanical pleurodesis. From a total of 73 chemical pleurisy, 44 (60.2%) patients were treated with tetracycline, and 29 (39.7%) patients were treated with bleomycin. Out of a total of 22 cases of mechanical pleurodesis, 7 cases underwent chemical pleurodesis in addition to chemical pleurodesis.

To classify the response to treatment in the studied patients, 2 main criteria were considered, and the necessary information was collected during a telephone interview. The first criterion for response to treatment was the lack of fluid re-accumulation and, as a result the improvement of clinical symptoms after pleurodesis. The second criterion was the number of complications after surgery and other necessary considerations.

After reviewing the files and contacting the patients, due to reasons such as non-response, non-cooperation, lack of necessary information, or the death of the patient, information was collected from a total of 61 cases out of 95 treated patients. Based on the classification using the first and second criteria, the patients were divided into 3 groups. The first group with a complete response did not suffer from re-accumulation of effusion fluid, and the complications of surgery were minimal. The second group with partial response means that fluid accumulation was not completely prevented, but respiratory symptoms improved to a large extent and did not cause serious complications, and the third group of patients who either needed to return due to insufficient response or severe complications after surgery. From a total of 61 patients; 46 patients were treated by chemical method and 15 patients by mechanical method. In the subset of chemical methods, 27 cases were using tetracycline, and 19 cases were using bleomycin. In patients treated with tetracycline, 15 cases were associated with complete response, 5 cases with partial response, and 7 cases with initial non-response. In patients treated with bleomycin, 10 cases had a complete response, 4 cases had a partial response, and 5 cases had no initial response. In patients treated with a mechanical method, 10 patients had a complete response, 2 patients had a partial response, and 3 patients had no initial response. Almost all patients complained of local pain after surgery. In 12 patients, fever and pain at the surgical site were mentioned. Mild gastrointestinal symptoms were reported in 6 patients following surgery, and empyema was reported in 1 patient who had an immunodeficiency background.

According to the above table, 74% of patients responded to treatment using the tetracycline chemical method, 73.6% using the bleomycin chemical method, and 79.9% using the mechanical method.

## 4 DISCUSSION

The objective of our study was to comprehensively examine the characteristics, outcomes, and treatment responses of patients with chronic pleural effusion. According to the latest studies and sources, the most common causes of chronic pleural effusion in the world are congestive heart failure, then various malignancies, and finally, liver failure. In our patient population, malignancies were identified as the predominant cause of chronic pleural effusion, which is consistent with the referral pattern to our center.

One noteworthy aspect that sets our study apart is the higher prevalence of malignant effusion in younger patients. These findings challenge conventional expectations and underscore the importance of considering malignancy, even in demographics where it may not be the primary consideration. Early detection and management strategies in younger patients are crucial, as highlighted by the need for further investigation into specific malignancies affecting this age group and their variations compared to global patterns.

Regarding the types of malignancies identified, our study found a notable preference for adenocarcinoma among lung malignancies. We recognize the potential interest in exploring the differences between these malignancies in our population compared to other countries. It is essential to acknowledge the limitation of our study concerning the lack of long-term follow-up data. While we presented the immediate response rates, including recurrence rates and follow-up durations, it would offer a more comprehensive understanding of the durability of these treatment outcomes.

Furthermore, investigating any variations in the distribution of other types of malignancies within our study cohort can offer a comprehensive understanding of the spectrum of malignancies contributing to chronic pleural effusion. Future studies with extended follow-up periods are necessary to assess the long-term efficacy of these methods, particularly recurrence rates over time.

By emphasizing the novel findings related to age-specific prevalence and malignancy types, we aim to enhance the relevance of our study in the broader context of global research on pleural effusion.

In the chemical method, after draining the effusion, a sucrose-giving substance (such as tetracycline and bleomycin) is injected into the pleural space using thoracocentesis or thoracoscopy [12, 13].

Recommendations in favor of using ultrasound to guide pleural interventions, not performing pleural interventions in patients without MPE symptoms, and using pleural catheters or chemical pleurodesis in patients with MPE symptoms and suspected expandable lung are very important. Following these recommendations will lead to fewer hospitalizations and better patient outcomes [14, 15]. Researchers want to note that MPE surgical options in the form of thoracic abrasion may be equivalent to chemical pleurodesis in certain scenarios, according to studies [16]. In addition, several other areas of interest have not been fully elucidated [17]. There are not enough studies to compare palliative methods of MPE with antitumor treatment [18].

Considering that MPE is mostly in the late stages of malignant diseases, currently, methods are still more focused on symptom management. It is true that for the most effective treatment, clinicians must be able to detect the underlying disease at an early stage (*e.g*., breast cancer public health interventions, screening programs, *etc*.) [19, 20]. Another area of interest with high-quality evidence is the management of trapped lungs in MPE. Researchers believe that using IPC in such an environment is valuable [21, 22]. However, the use of other methods (for example, the use of intrapleural fibrinolytic therapy) may also be useful in certain cases [23]. In addition, there is still no consensus on the MPE drainage volume. Traditionally, in the therapeutic setting, fluid removal was stopped when the total amount of fluid removed reached 1000 to 1500 mL due to the fear of re-expansion of pulmonary edema and pneumothorax. Ault *et al*. showed that common assumptions about thoracocentesis safety guidelines are usually incorrect [24].

Researchers believe that currently, no maximum volume of fluid can be safely removed during therapeutic thoracocentesis. Decisions regarding the amount of fluid removed should remain within the domain of experienced clinicians [25]. In the present study, out of 95 patients with MPE (malignant pleural effusion), 73 patients (76.8%) were treated with chemical pleurodesis, and 22 patients (23.1%) were treated with mechanical methods. After accessing 61 cases of treated patients, it was found that the mechanical method was 79.9% effective, and the chemical method was 73.8% effective [26, 27]. Future studies with extended follow-up periods are necessary to assess the long-term efficacy of these methods, particularly recurrence rates over time.

The choice between chemical and mechanical pleurodesis was based on the patient’s clinical condition and the physician’s judgment. Chemical pleurodesis was preferred for less invasive treatment, with tetracycline or bleomycin selected based on availability, cost, and physician experience. Mechanical pleurodesis was used when stronger adhesion was needed or when chemical methods had failed. It was sometimes combined with chemical agents to reduce the recurrence of effusions. One of the key findings of our study was the prevalence of MPE among patients with chronic pleural effusion. We found that out of the 144 patients included in the study, 95 patients (66%) had MPE. This highlights the critical need for early identification and treatment strategies, as MPE is frequently associated with late-stage malignancies, significantly impacting patients' quality of life.

Regarding demographic characteristics, we observed that the average age of the patients with chronic pleural effusion was 47.59 years, with a standard deviation of 12.13. This finding aligns with the existing literature, which suggests that pleural effusion is more commonly seen in middle-aged and older individuals (reference). However, we did not find a significant difference when comparing the average age of patients with MPE and other patients. This suggests that age alone may not be a reliable predictor of MPE development in patients with chronic pleural effusion.

The analysis of laboratory findings provided valuable insights into the diagnostic markers associated with MPE. Our results showed a significantly higher number of White Blood Cells (WBC) in patients with MPE compared to those without MPE. This finding aligns with previous studies that have demonstrated the role of elevated WBC count as an indicator of malignancy in pleural effusion (reference). Additionally, we observed a higher Erythrocyte Sedimentation Rate (ESR) and Lactate Dehydrogenase (LDH) level in patients with MPE.

The analysis of pleural fluid characteristics revealed significant differences between patients with MPE and other patients. Patients with MPE had higher levels of protein and LDH in their pleural fluid compared to those without MPE. These findings are consistent with the exudative nature of MPE, characterized by increased pleural fluid protein and LDH levels (reference). Moreover, the presence of lymphocytes in the pleural fluid was significantly higher in patients with MPE.

The prevalence analysis of the type of pleural effusion demonstrated a significant association between the presence of MPE and exudative pleural effusion. A large majority (90.9%) of patients with MPE had an exudative pleural effusion, while only a minority (11.1%) of other patients had the same type. This finding further supports the role of pleural fluid analysis, including protein and LDH levels, in differentiating MPE from other causes of pleural effusion.

The clinical implications of our study findings are noteworthy. Firstly, the high prevalence of MPE among patients with chronic pleural effusion emphasizes the importance of early consideration and evaluation for malignancy in such cases. Prompt diagnosis and appropriate management strategies, such as pleurodesis, can significantly improve patient outcomes and quality of life. Secondly, the diagnostic markers identified in this study, including elevated WBC count, ESR, LDH levels, and lymphocyte presence in pleural fluid, can aid in the identification of MPE and guide clinical decision-making. These markers can contribute to a more targeted and efficient diagnostic approach in patients with pleural effusion. The high prevalence of lymphocyte-predominant exudative pleural effusions among MPE patients further emphasizes the utility of pleural fluid analysis in differentiating malignant from non-malignant causes.

Finally, given the high malignancy rate in this cohort, it is essential to explore region-specific variations in cancer epidemiology and treatment responses. These insights can contribute to the development of tailored strategies that address the unique needs of patients in different demographic and geographic settings. The comparison highlights the clinical importance of distinguishing effusion etiologies and tailors diagnostic and therapeutic approaches accordingly. By emphasizing these differences, our study contributes to the growing body of evidence supporting a targeted diagnostic algorithm for pleural effusions, ensuring timely and accurate management.

## CONCLUSION

The results of the present study showed that the most common causes of chronic pleural effusion include malignancies and chronic heart failure, respectively. The most common malignancies included lung malignancies with a preference for adenocarcinoma. By analyzing the amount of protein, LDH, and the number of WBCs and their differentiation in the pleural fluid, it is possible to distinguish malignant pleural effusion from other causes of chronic pleural effusion. Using chemical agents’ tetracycline, bleomycin, and mechanical pleurodesis has greatly improved the symptoms of MPE patients and prevented fluid re-accumulation.

Based on our findings, we recommend comprehensive cytological and biochemical examinations in cases of chronic effusion to improve diagnostic accuracy and guide treatment strategies more effectively. This approach aligns with the evidence presented in our study and could help identify underlying conditions and optimize patient management.

The main limitation of the present study was the lack of access to patients, which was addressed through telephone contact, laboratory data, and patient records.

It is recommended to design and conduct a multicenter cohort study with long-term patient follow-up to evaluate the response to treatment.

## Figures and Tables

**Table 1:** Comparison of average laboratory findings of patients with MPE (malignant pleural effusion) and other patients.

Signs/MPE	No	Yes	Statistical Hypothesis Tests	*p*-value
Blood and serum	Mean (SD)	Mean (SD)	-	-
WBC	771.39 (342.22)	11978.4 (476.62)	Independent T	0.0001
Hb	11.77 (3.12)	11.25 (3.07)	Mann-Whitney	0.276
ESR	21.86 (9.65)	38.94 (10.89)	Independent T	0.0001
Protein	6.40 (0.76)	6.54 (0.73)	Independent T	0.276
LDH	564.25 (90.16)	661.59 (82.70)	Independent T	0.0001
Pleural effusion	-	-	-	-
Protein	2.52 (0.75)	4.21 (0.92)	Independent T	0.0001
LDH	322.68 (95.18)	542.76 (88.13)	Independent T	0.0001
Glucose	114.16 (17.57)	112.66 (16.70)	Independent T	0.599
WBC	492.89 (91.83)	5224.03 (1207.23)	Independent T	0.0001
Lymphocyte %	30.22 (15.01)	78.12 (11.07)	Independent T	0.0001

**Table 2:** Prevalence of type of pleural effusion in patients with MPE (malignant pleural effusion) and other patients.

Type/MPE	No (Percent)	Yes	*p*-value
Exudate	(9%)9	(90.9%)90	0.0001
Transudate	(88.8%)40	(11.1%)5	
Total	(100%)49	(100%)95	

## Data Availability

Data sharing is not applicable to this article as no datasets were generated or analyzed during the current study.

## LIST OF ABBREVIATIONS

WBC: = White Blood Cell
ESR: = Erythrocyte Sedimentation Rate
MPE: = Malignant Pleural Effusion
STROCSS: = Strengthening the Reporting of Cohort Studies in Surgery
LDH: = Lactate Dehydrogenase

## STANDARDS OF REPORTING

STROBE and STROCSS guidelines were followed.
